# Comparative Analyses of Physiological Responses of *Cynodon dactylon* Accessions from Southwest China to Sulfur Dioxide Toxicity

**DOI:** 10.1155/2014/916595

**Published:** 2014-07-06

**Authors:** Xi Li, Ling Wang, Yiqiao Li, Lingxia Sun, Shizhen Cai, Zhuo Huang

**Affiliations:** ^1^College of Landscape Architecture, Sichuan Agricultural University, No. 211 Huimin Road, Wenjiang, Sichuan 611130, China; ^2^Business School, Sichuan Agricultural University, Dujiangyan, Sichuan 611830, China

## Abstract

Sulfur dioxide (SO_2_), a major air pollutant in developing countries, is highly toxic to plants. To achieve better air quality and landscape, planting appropriate grass species in severe SO_2_ polluted areas is very critical. *Cynodon dactylon*, a widely used warm season turfgrass species, has good SO_2_-tolerant ability. In this study, we selected 9 out of 38 *C. dactylon* accessions from Southwest China as representatives of high, intermediate SO_2_-tolerant and SO_2_-sensitive accessions to comparatively analyze their physiological differences in leaves under SO_2_ untreated and treated conditions. Our results revealed that SO_2_-tolerant *C. dactylon* accessions showed higher soluble sugar, proline, and chlorophyll a contents under both SO_2_ treated and untreated conditions; higher chlorophyll b and carotenoid under SO_2_ treated condition; lower reactive oxygen species (ROS) level, oxidative damages, and superoxide dismutase (SOD) activities under SO_2_ treated condition; and higher peroxidase (POD) activities under SO_2_ untreated condition. Further results indicated that SO_2_-tolerant *C. dactylon* accessions had higher sulfur contents under both SO_2_ treated and untreated conditions, consistent with higher SO activities under both SO_2_ treated and untreated conditions, and higher SiR activities under SO_2_ treated condition. Taken together, our results indicated that SO_2_ tolerance of *C. dactylon* might be largely related to soluble sugar, proline and chlorophyll a contents, and SO enzyme activity.

## 1. Introduction

Sulfur dioxide (SO_2_), a gaseous pollutant with bad odor in the atmosphere, is mainly emitted from anthropogenic sources. It is estimated that more than 70% of global SO_2_ is emitted from anthropogenic sources, half of which is from combustion of fossil fuel [1]. With rapid development of economy in developing countries, emission of SO_2_ into the atmosphere has been increasing quickly. As the biggest developing country in the world, China is leading the world as the biggest SO_2_ emitter, contributing to about one-fourth of the global emission and more than 90% of East Asia emission since the 1990s [2]. Total SO_2_ emission in China increased by 53%, from 21.7 Tg (1 Tg = 10^12^ g) in 2000 to 33.2 Tg in 2006, at an annual growth rate of 7.3%. The SO_2_ emission began to decrease after 2006 mainly due to the widespread application of flue-gas desulfurization (FGD) devices at all newly built thermal power units in order to implement a comprehensive national policy strategy of energy conservation and emission reduction since 2005. However, the total SO_2_ emissions are still very high (27.7 Tg) in 2010 due to the dramatic growth of industrial production and energy consumption [3]. Thereafter, high level of SO_2_ in the atmosphere will be a major concern in developing countries in a long period of forthcoming time.

In the atmosphere, when gaseous SO_2_ meets with water, considerable amounts of SO_2_ are converted to sulphurous acid, which is the important component of acid rain. Sulfur is well known to be a basic constituent of sulfur-containing amino acids, iron-sulfur clusters, cofactors, polysaccharides, and lipids for all living organisms. SO_2_ can enter plants via their stomata by the process of photosynthesis and respiration [4]. Plant has the ability to incorporate this kind of inorganic sulfur into sulfur-containing amino acids, proteins, and glutathione (GSH) and sulfur can also serve as the sulfur precursor of sulfur-containing secondary products in plant. However, above a certain threshold, both SO_2_ and acid rain are highly toxic to plants, causing many visible symptoms in the plant like yellowing, chlorosis, bleaching, and even killing foliage depending on the dosages [4]. Because of the harmful effects of SO_2_, some plants cannot grow robustly and even die in severe polluted urban or industrial districts, creating “dead zones” without greenery. To achieve better air quality and landscape effect in such polluted areas, the plants with high resistance to SO_2_ should be selected out for use. Tree species tolerant to SO_2_ were selected out or developed for planting in air polluted areas [5–7].

Turfgrasses were extensively used in a sole manner or in combination with trees for environmental greening. Importantly, grass plants are more resistant to SO_2_ than woody plants, because the former have a higher S : C ratio than the latter and therefore can take up more SO_2_ from the atmosphere [8]. Turfgrasses can be generally classified as cool season, warm season, or evergreen types. A few studies on tolerance to SO_2_ of cool-season grass populations in polluted areas have been carried out in the past decades. These studies mainly focused on identification of tolerant populations from cool-season species of* Dactylis glomerata, Festuca rubra, Holcus lanatus, Lolium perenne,* and* Phleum bertolonii* [9]; comparison of stomatal morphology and resistance, membrane permeability, and the uptake and metabolism of ^35^SO_3_ and ^35^SO_2_ in cool-season species of D.* glomerata*,* F. rubra*,* H. lanatus*, and* L. perenne* [10]; investigation on the rate of development of tolerance in cool-season species of* F. rubra*,* L. multiflorum*,* L. perenne*,* P. pratense,* and* Poa pratensis* [11]; and genetic nature of tolerance in cool-season species of* L. perenne* [12]. In such studies,* Cynodon dactylon*, a warm season perennial grass species, is not included, which is widely used as turfgrass on sports fields, golf courses, roadsides, and lawns in city or industry districts in warm season. Recently, our comparative study on physiological and growth performances found that* C. dactylon* displayed the highest resistance to SO_2_ among four warm season turfgrasses including* C. dactylon*,* Eremochloa ophiuroides*,* Paspalum notatum*, and* Zoysia japonica* [13]. In the present study, we firstly compared influences of SO_2_ on leaves of 38 wild* C. dactylon* accessions from Southwest China. Based on injury rate of SO_2_ to leaves, nine* C. dactylon* accessions representing high SO_2_-tolerant, intermediate SO_2_-tolerant, and SO_2_-sensitive to SO_2_ accessions were selected to comparatively study relationships between SO_2_ tolerance and several physiological parameters. This study gained some insights into understanding the genetic and molecular mechanisms of* C. dactylon* to SO_2_ and provided guideline for selection and development of* C. dactylon* variations for planting in SO_2_ polluted urban or industrial areas.

## 2. Materials and Methods

### 2.1. Plant Materials and Growth Conditions

**Figure 1:**
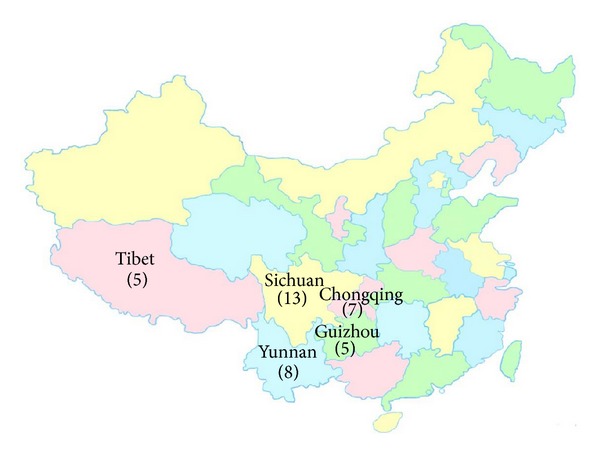
The collection sites of the 38 samples of wild* C. dactylon *in Southwest China. Provinces where samples were collected are illustrated with number of* C. dactylon *accessions in parentheses underneath in map.

Thirty-eight wild* C. dactylon *accessions used in this study were sampled from Sichuan Province, Chongqing municipality, Yunnan Province, Guizhou Province, and Tibet Autonomous Region in Southwestern China between years 2011 and 2012. A complete list of accession descriptions and geographical origins was provided in Table 1 and Figure 1. The wild* C. dactylon* accessions were collected originally from roadside, riverside, floodland, fieldridge, wasteland, hillside, or city park. All wild accessions used in this study were determined to be* C. dactylon* based on morphological characteristics as described by Harlan [14].

The experiments were carried out between April and August, 2013, at Experimental Station of Grass Science, Sichuan Agricultural University, Ya'an, Sichuan Province, China. The experimental location is 600 m in altitude with a humid subtropical climate. Mean annual precipitation, annual temperature, and relative air humidity in the area are 1800 mm, 16.2°C, and 79%, respectively. All the* C. dactylon* accessions were planted in plastic pots (18 cm in top diameter, 14 cm in bottom diameter, and 15 cm in depth) filled with typical sandy loam soil in the local place in April. Each* C. dactylon* accession was replicated six times. All* C. dactylon* grasses were grown under natural conditions for 2 months with regular watering every day and fertilizing and cutting every four weeks prior to the experimental treatment.

### 2.2. Stress Treatment and Experimental Design

After two-month growth, three pots of grass plants with nearly the same crown from each accession were chosen from six replications (as mentioned above) for SO_2_ stress treatment. All of the selected pots of grass plants were fumigated with SO_2_ at a concentration of 3.75 mg/L in a custom-made fumigation chamber (85 cm × 85 cm × 40 cm) for 3 h per day over 7 days as described in our previous study [13]. The day when SO_2_ fumigation started was designated as day 0. In order to achieve a uniform environment in the chamber, a fan was attached to the chamber ceiling to mix the SO_2_. A SO_2_ gas detector (Z-1300, Environmental Sensors Co., Boca Raton, FL, USA) was used to measure the concentration of SO_2_ and to keep the gas concentration constant in the chamber during the experiment. After fumigation treatment, grass plants were taken out and grown under natural conditions with regular watering every day. The remaining three pots of grass plants without SO_2_ treatment from each accession served as control. Based on injury rate of SO_2_ to leaves after 7-day treatment of SO_2_, three high SO_2_-tolerant, three intermediate SO_2_-tolerant, and three SO_2_-sensitive* C. dactylon* accessions were selected out for physiological studies. Leaves 2 cm above the soil from* C. dactylon* plants treated by SO_2_ after 7 days were collected and brought back to laboratory for analysis. Leaves from* C. dactylon* plants without SO_2_ treatment at day 0 served as control.

### 2.3. Measurement of Total Soluble Sugars

The total soluble sugars were determined using the anthrone method as previously described by Lu et al. [15] with some modifications. Briefly, 0.2 mg dried leaf samples were extracted in 5 mL of 80% (v/v) ethanol at 80°C for 40 min and centrifuged at 15,000 ×g for 10 min. The pellets were further extracted twice with another 5 mL of 80% (v/v) ethanol. The supernatants were combined together and depigmented by activated charcoal at 80°C for 30 min. For the determination of soluble total sugars, 0.2 mL of the filtrate was mixed with 3 mL of 0.15% (w/v) anthrone reagent (0.3 g anthrone was dissolved in 200 mL of 7.74 M H_2_SO_4_) and then heated at 90°C for 20 min. Finally, soluble total sugar level was determined at 620 nm of absorbance using a UV/VIS spectrophotometer Model 723PC (Jinghua Instruments, Shanghai, China).

### 2.4. Measurement of Proline Content

Proline content was estimated according to the method based on proline's reaction with ninhydrin described by Bates et al. [16] with modification. Briefly, 0.2 g leaf samples were ground in 5 mL 3% (w/v) sulfosalicylic acid and then filtered through 0.45 *μ*m filter paper. Two microliters of filtrate was mixed with equal volumes of ninhydrin reagent and glacial acetic acid. Well mixed solutions were boiled at 100°C for 1 h. The reaction was terminated in an iced bath and the chromophore was extracted with 4 mL toluene and its absorbance at 520 nm was determined using a UV/VIS spectrophotometer Model 723PC (Jinghua Instruments, Shanghai, China).

### 2.5. Estimation of Chlorophyll and Carotenoid

Photosynthetic pigments from the leaves were extracted as described by Lichtenthaler and Wellburn [17] with modification. Leaf samples (~0.2 g) were ground in 2 mL of 80% acetone and ethyl alcohol (1 : 1), using a mortar and pestle, and then filtered through 0.45 *μ*m filter paper. Absorbance of the resulting extracts was measured at three wavelengths 663, 646, and 470 nm for chlorophyll a, chlorophyll b, and carotenoids, respectively, using a UV/VIS spectrophotometer Model 723PC (Jinghua Instruments, Shanghai, China). The amounts of pigments were calculated according to the equations developed by Lichtenthaler and Wellburn [17]. Total chlorophyll was obtained from the sum of chlorophylls a + b.

### 2.6. Determination of H_2_O_2_ Level

For grass protein extraction, about 0.2 g fresh leaves were ground with liquid nitrogen and then homogenized in extraction buffer (50 mM sodium phosphate buffer, pH 7.8). After centrifugation at 15,000 ×g for 15 min at 4°C, the supernatant was used for determination of H_2_O_2_ levels as described by Hu et al. [18]. Briefly, 1 mL of the supernatant was mixed with 1 mL of 0.1% titanium sulphate in 20% H_2_SO_4_ (v/v) thoroughly for 10 min. After being centrifuged at 15,000 ×g for 10 min at room temperature, the absorbance of the supernatant was measured at 410 nm using a UV/VIS spectrophotometer Model 723PC (Jinghua Instruments, Shanghai, China).

### 2.7. SOD, POD, CAT, SiR, and SO Enzyme Assays

Fresh leave sample (~0.5 g) was homogenized in 5 mL of 0.1 M phosphate buffer (pH 6.8) containing 1 mM EDTA, 1 mM dithiothreitol, and 2% (w/v) polyvinylpyrrolidone (PVP) using a chilled mortar and pestle on ice. The homogenate was centrifuged at 15,000 ×g for 15 min at 4°C, and the supernatant was used for enzyme activity. Soluble protein content was determined following the Bradford method [19] with BSA as standard. Superoxide dismutase (SOD) activity was determined spectrophotometrically at 560 nm based on the measurement of inhibition in the photochemical reduction of nitroblue tetrazolium (NBT) [20, 21]. Peroxidase (POD) activity was determined by the guaiacol oxidation method [22]. Catalase (CAT) activity was determined by measuring the rate of decomposition of H_2_O_2_ at 240 nm, as described by Aebi [23]. Sulfite reductase (SiR) activity was estimated by the coupled SiR/OASTL assay [24, 25] with the addition of NADPH and tungstic acid [26]. Sulfite oxidase (SO) activity was determined by measuring sulphite disappearance using OH- mediated discolouring of fuchsine according to Pachmayr's report [27].

### 2.8. Estimation of MDA Content

Malondialdehyde (MDA) content was determined using the method described by Fu and Huang [28]. Fresh leaf sample (0.2 g) was homogenized with 2 mL of 0.1% (w/v) trichloroacetic acid (TCA) using a chilled mortar and pestle on ice. The homogenate was centrifuged at 15,000 ×g for 20 min, at 4°C, and the supernatant was used for lipid peroxidation analysis. A total of 4 mL of 0.5% thiobarbituric acid (TBA) in 20% TCA was added to 1 mL of the supernatant. The mixture was incubated in hot water (95°C) for 30 min and cooled immediately on ice to stop the reaction and centrifuged at 15,000 ×g for 20 min. Absorbance was measured at 532 and 600 nm, and MDA concentration was estimated by subtracting the nonspecific absorption at 600 nm from the absorption at 532 nm.

### 2.9. Estimation of Sulfur Content

For sulfur (S) determination, the turbidimetric method described by Reyes-Díaz et al. [29] was applied. Biomass of whole plant dried for 48 h was treated with 95% magnesium nitrate and ashed at 500°C for 8 h. Then the ashed samples were digested in 10 mL of 2 M HCl at 150°C for 60 min. After addition of barium chloride (BaCl_2_) and Tween-80 into the solution, its absorbance was immediately measured using a UV/VIS spectrophotometer Model 723PC (Jinghua Instruments, Shanghai, China) at 440 nm.

### 2.10. Statistical Analysis

All experiments in this study were repeated at least three times. Statistical analysis (mean ± standard error) was performed and chart was created using relative tools of Microsoft Excel 2010. All data were analyzed by ANOVA using SPSS 13.0 software package (SPSS Inc., Chicago, USA), and then LSD method was used to detect possible differences among the accessions. Asterisk symbols above the columns in the figures indicate significant differences at *P* < 0.05 (Student's *t*-test).

## 3. Results

### 3.1. Leaf Injury under SO_2_ Stress Condition

**Figure 2:**
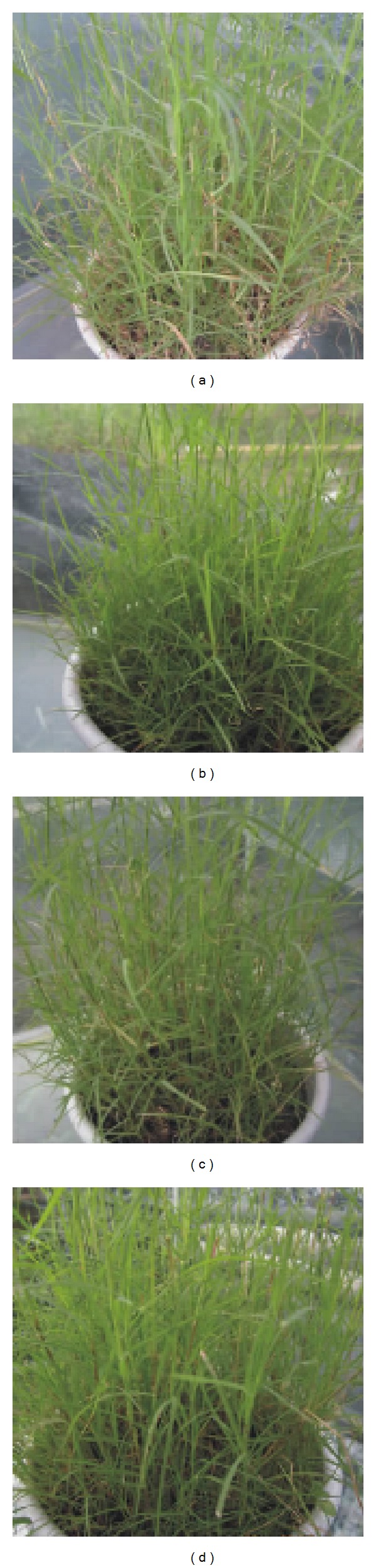
Symptoms of* C. dactylon *accessions in response to SO_2_. (a) SO_2_-sensitive representative* C. dactylon *accession CQ1116, (b) intermediate SO_2_-tolerant representative* C. dactylon *accession SC1217, (c) high SO_2_-tolerant representative* C. dactylon *accession SC1203, and (d)* C. dactylon *accession CQ1116 without SO_2_ treatment as a control.

After 7-day fumigation treatment by SO_2_, injury symptoms appeared on leaves of all the 38* C. dactylon* accessions. The visible symptoms consisted of bifacial, marginal, or interval necrosis and chlorosis on leaves at the full stage of development (Figure 2). The necrotic areas ranged from white to brown in color, and the margins of the necrotic areas are mostly irregular and occasionally dark in color. Injury rate of leaves varied in* C. dactylon* accessions from 38.3% in accession YN1205 to 13.3% in accession SC1203 (Table 2). It seemed that accessions originated from city park and hillside had higher SO_2_ tolerance than other habitat origins (Tables 1 and 2). To further study the physiological response of* C. dactylon* to SO_2_, we selected three accessions of SC1203, SC1209, and GZ1110 as high SO_2_-tolerant representatives, three accessions of SC1217, YN1110, and XZ1206 as intermediate SO_2_-tolerant representatives, and three accessions of YN1205, CQ1116, and SC1208 as SO_2_-sensitive representatives based on the injury rate of leaves and the geographic distribution (Tables 1 and 2).

### 3.2. Changes of Sugar and Proline under SO_2_ Stress Condition

**Figure 3:**
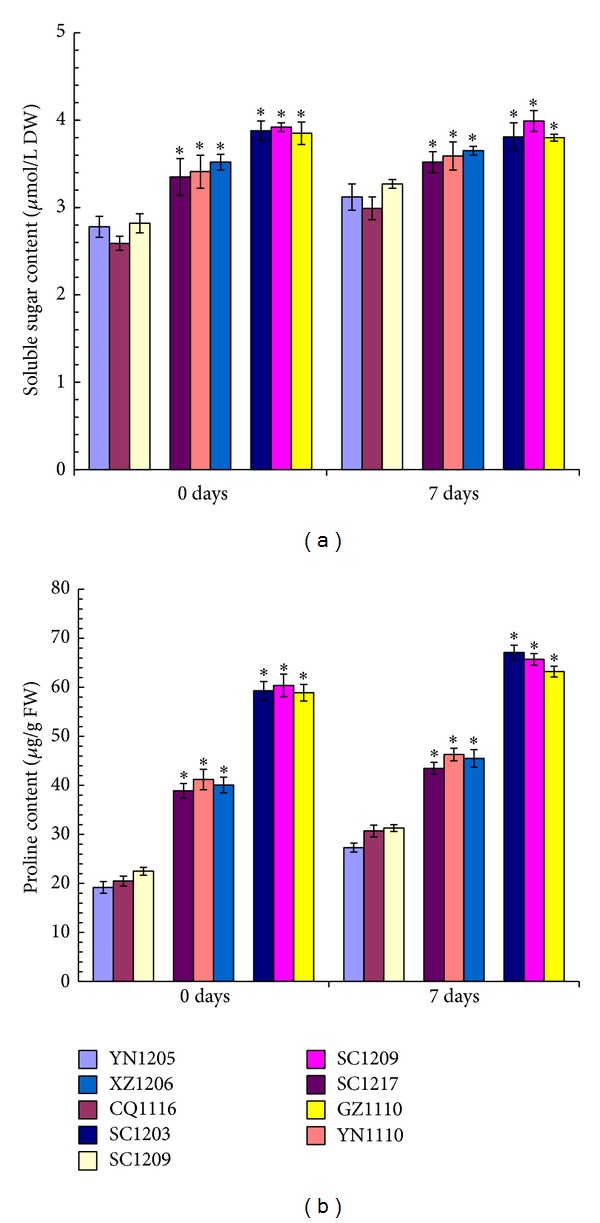
Comparison of soluble sugar (a) and proline (b) in leaves from nine selected* C. dactylon *accessions in response to SO_2_ stress. Mean values are presented with vertical error bars representing the standard deviations (*n* = 3). The asterisk symbols indicate significant differences between SO_2_-sensitive accessions and SO_2_-tolerant accessions.

The soluble sugar and proline contents in leaves from all of the nine* C. dactylon* accessions increased along with the increase of their SO_2_ tolerability (Figure 3). Moreover, the soluble sugar and proline contents from all of the high SO_2_-tolerant* C. dactylon* accessions and intermediate SO_2_-tolerant* C. dactylon* accessions were significantly higher than those from any of the three SO_2_-sensitive* C. dactylon* accessions at both 0-day time-point without SO_2_ treatment and 7-day time-point after SO_2_ fumigation treatment. However, the soluble sugar and proline contents from 7-day time-point after SO_2_ fumigation treatment showed no significant change when they were compared with those from 0-day time-point in any* C. dactylon* accession, which indicates that both soluble sugar and proline are not induced or inhibited in* C. dactylon* under SO_2_ stress condition (Figure 3).

### 3.3. Changes of Photosynthetic Pigments under SO_2_ Stress Condition

**Figure 4:**
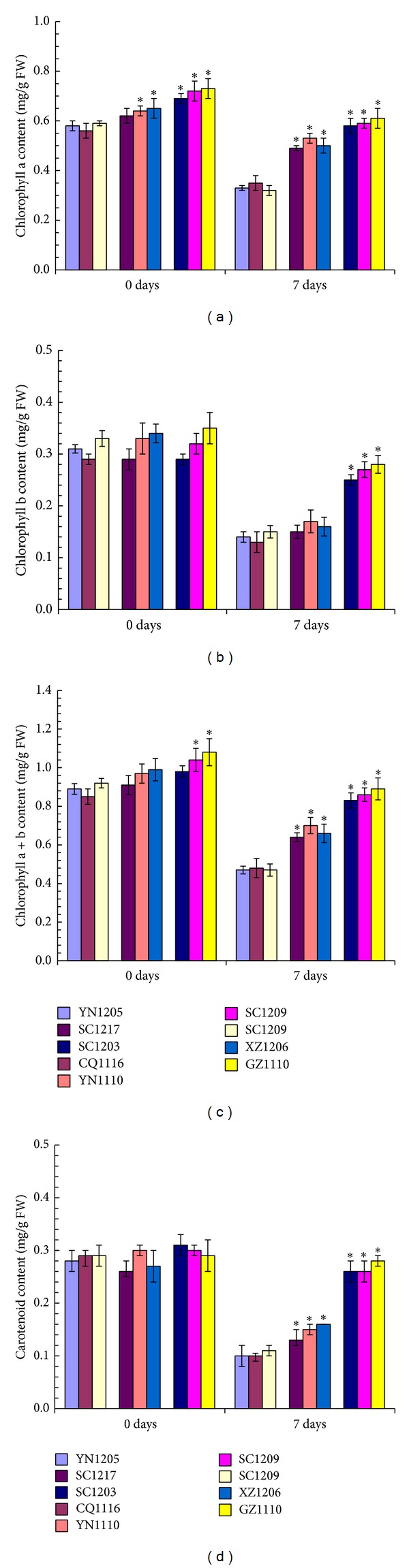
Comparison of chlorophyll a (a), chlorophyll b (b), total chlorophyll (c), and carotenoid (d) in leaves from nine selected* C. dactylon *accessions in response to SO_2_ stress. Mean values are presented with vertical error bars representing the standard deviations (*n* = 3). The asterisk symbols indicate significant differences between SO_2_-sensitive accessions and SO_2_-tolerant accessions.

Contents of photosynthetic pigments in leaves from all of the nine* C. dactylon* accessions decreased under SO_2_ stress condition but showed different patterns with different pigment (Figure 4). Chlorophyll a contents from two intermediate SO_2_-tolerant* C. dactylon* accessions (YN1110 and XZ1206) and from all of the three high SO_2_-tolerant* C. dactylon* accessions were significantly higher than those from any of SO_2_-sensitive* C. dactylon* accessions in an increasing trend along with the increase of SO_2_ tolerability at 0-day time-point (Figure 4(a)). Under SO_2_ stress condition, chlorophyll a contents in leaves from intermediate and high SO_2_-tolerant* C. dactylon* accessions reduced significantly less than those from SO_2_-sensitive* C. dactylon* accessions. No significant differences of chlorophyll b contents were observed among the high SO_2_-tolerant, intermediate SO_2_-tolerant, and SO_2_-sensitive* C. dactylon* accessions at 0-day time-point (Figure 4(b)). After 7-day stress treatment by SO_2_ fumigation, chlorophyll b contents reduced in leaves from all of the nine* C. dactylon* accessions. The contents of chlorophyll b showed no significant differences between intermediate SO_2_-tolerant and SO_2_-sensitive* C. dactylon* accessions, but significantly less reduction of chlorophyll b content was observed in high SO_2_-tolerant* C. dactylon* accessions. As for total chlorophyll content, it showed a similar pattern with chlorophyll a in leaves from all of the nine* C. dactylon* accessions (Figure 4(c)). Carotenoid contents showed no significant differences among the high SO_2_-tolerant, intermediate SO_2_-tolerant, and SO_2_-sensitive* C. dactylon* accessions at 0-day time-point but significantly less reduced along with the increase of SO_2_ tolerability of* C. dactylon* accessions after 7-day SO_2_ stress treatment (Figure 4(d)).

### 3.4. Changes of ROS Level and Antioxidant Enzyme Activities under SO_2_ Stress Condition

**Figure 5:**
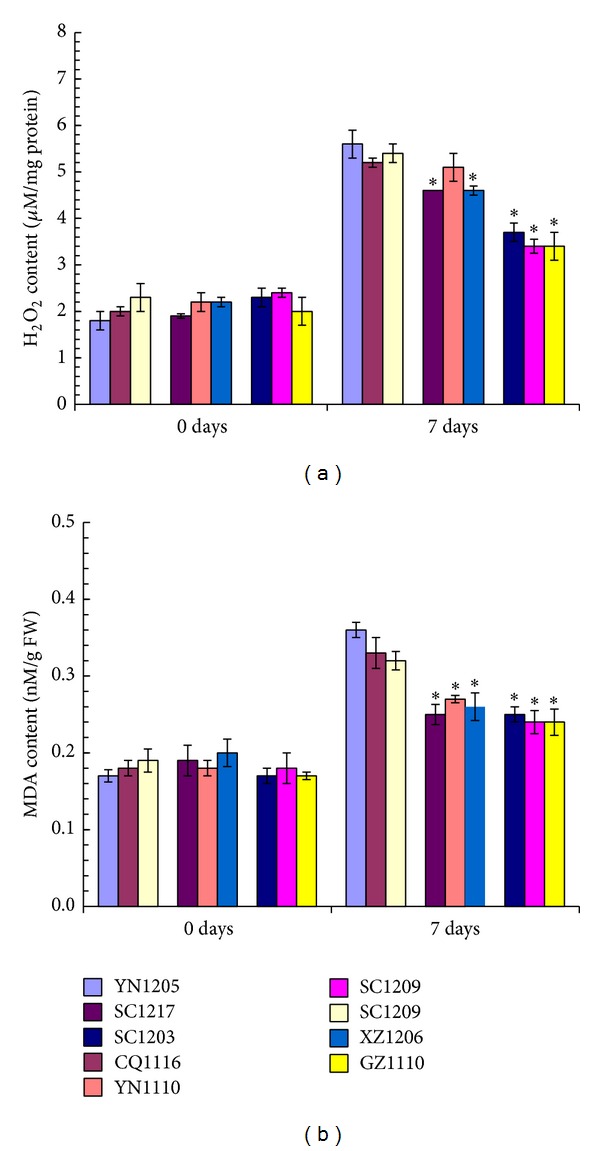
Comparison of ROS levels reflected by H_2_O_2_ (a) and MDA (b) contents in leaves from nine selected* C. dactylon *accessions in response to SO_2_ stress. Mean values are presented with vertical error bars representing the standard deviations (*n* = 3). The asterisk symbols indicate significant differences between SO_2_-sensitive accessions and SO_2_-tolerant accessions.

As two major indicators for reactive oxygen species (ROS) level and oxidative damage, hydrogen peroxide (H_2_O_2_) and malondialdehyde (MDA) contents were tested in this study. As shown in Figure 5, the high SO_2_-tolerant, intermediate SO_2_-tolerant, and SO_2_-sensitive* C. dactylon* accessions displayed nearly the same levels of H_2_O_2_ and MDA in leaves at 0-day time-point without SO_2_ treatment (Figures 5(a) and 5(b)). After 7-day SO_2_ fumigation treatment, levels of both H_2_O_2_ and MDA increased in leaves from all of the nine* C. dactylon* accessions. When compared within all of the nine* C. dactylon* accessions, levels of both H_2_O_2_ and MDA in leaves from high SO_2_-tolerant* C. dactylon* accessions and intermediate SO_2_-tolerant* C. dactylon* accessions were significantly lower than those from SO_2_-sensitive* C. dactylon* accessions (Figures 5(a) and 5(b)).

**Figure 6:**
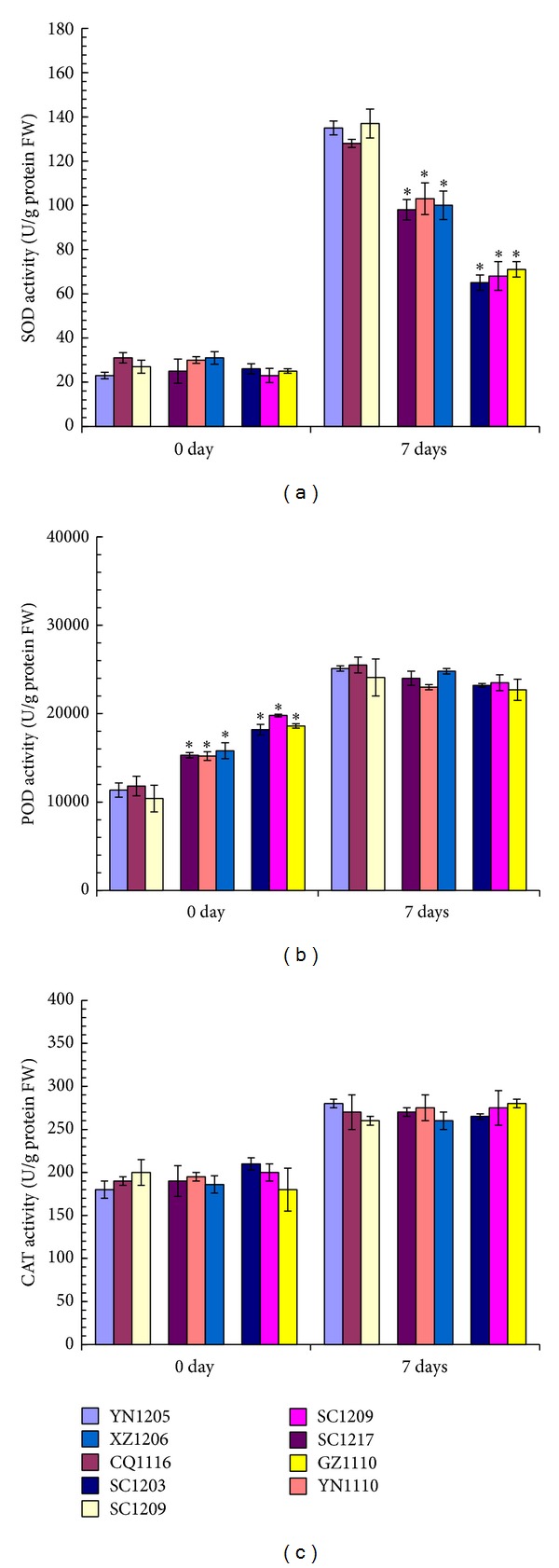
Comparison of antioxidant enzyme activities in leaves from* C. dactylon *accessions in response to SO_2_ stress. Antioxidant enzyme activities of SOD (a), POD (b), and CAT (c) in leaves from nine selected* C. dactylon *accessions in response to SO_2_ stress were compared. Mean values are presented with vertical error bars representing the standard deviations (*n* = 3). The asterisk symbols indicate significant differences between SO_2_-sensitive accessions and SO_2_-tolerant accessions.

To address the relationship between the changes of ROS level and the antioxidant enzyme activities, three major antioxidant enzymes, including SOD, POD, and CAT, were analyzed for their enzyme activities. SOD activities showed no significant differences (about 30 U/g protein FW) in leaves from high SO_2_-tolerant* C. dactylon* accessions, intermediate SO_2_-tolerant* C. dactylon* accessions, and SO_2_-sensitive* C. dactylon* accessions at 0-day time-point without SO_2_ treatment (Figure 6(a)). After 7-day SO_2_ stress treatment, SOD activities increased greatly in leaves from all of the nine* C. dactylon* accessions. However, the increase degree was in a decreasing trend along with SO_2_ tolerability of the* C. dactylon* accessions, displaying highest activities in SO_2_-sensitive* C. dactylon* accessions (more than 120 U/g protein FW) and lowest activities in high SO_2_-tolerant* C. dactylon* accessions (more than 60 U/g protein FW) (Figure 6(a)). POD activities increased in leaves from all of the nine* C. dactylon* accessions after 7-day SO_2_ stress treatment, but no significant differences were observed within high SO_2_-tolerant* C. dactylon* accessions, intermediate SO_2_-tolerant* C. dactylon* accessions, and SO_2_-sensitive* C. dactylon* accessions (Figure 6(b)). However, we found that POD activities in leaves from SO_2_-tolerant* C. dactylon* accessions were significantly higher than those from SO_2_-sensitive* C. dactylon* accessions in an increasing trend along with an increase of SO_2_ tolerability at 0-day time-point without SO_2_ treatment, displaying nearly 1.4-fold increase (15433/11183 U/g protein FW) and 1.7-fold increase (18866/11183 U/g protein FW) of enzyme activities in intermediate SO_2_-tolerant and high SO_2_-tolerant* C. dactylon* accessions, respectively (Figure 6(b)). CAT activities increased in leaves from all of the nine* C. dactylon* accessions after 7-day SO_2_ stress treatment, but no significant differences were observed in leaves from high SO_2_-tolerant* C. dactylon* accessions, intermediate SO_2_-tolerant* C. dactylon* accessions, and SO_2_-sensitive* C. dactylon* accessions either after 7-day SO_2_ stress treatment or at 0-day without SO_2_ treatment (Figure 6(c)).

### 3.5. Changes of Sulfur Content, SiR, and SO Enzyme Activities under SO_2_ Stress Condition

**Figure 7:**
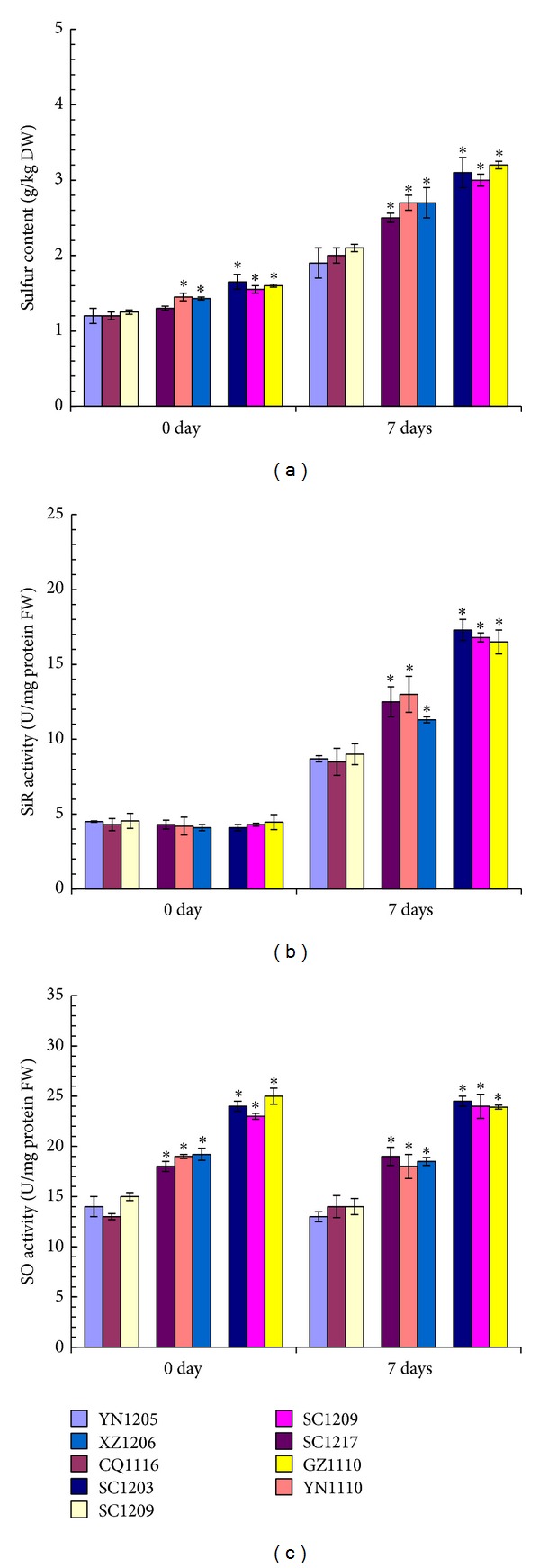
Comparison of sulfur contents (a), SiR activities (b), and SO activities (c) in leaves from nine selected* C. dactylon* accessions in response to SO_2_ stress. Mean values are presented with vertical error bars representing the standard deviations (*n* = 3). The asterisk symbols indicate significant differences between SO_2_-sensitive accessions and SO_2_-tolerant accessions.

Sulfur contents in leaves from two intermediate SO_2_-tolerant* C. dactylon* accessions (YN1110 and XZ1206) and all of the three high SO_2_-tolerant* C. dactylon* accessions were significantly higher than those from any of the SO_2_-sensitive* C. dactylon* accessions at 0-day time-point without SO_2_ stress treatment (Figure 7(a)). After 7-day SO_2_ fumigation treatment, sulfur contents increased in leaves from all of the nine* C. dactylon* accessions in an increasing trend along with increase of SO_2_ tolerability of the* C. dactylon* accessions. Moreover, sulfur contents in leaves from all of the high and intermediate SO_2_-tolerant* C. dactylon* accessions showed significantly higher levels than those from any of the SO_2_-sensitive* C. dactylon* accessions (Figure 7(a)). SiR activities were nearly in the same levels (about 5 U/mg protein FW) in leaves from all of the nine* C. dactylon* accessions at 0-day time-point without SO_2_ treatment (Figure 7(b)). After 7-day SO_2_ stress treatment, SiR activities increased about 2-fold (approximate 10 U/mg protein FW) in SO_2_-sensitive* C. dactylon* accessions, 2.4-fold (approximate 12 U/mg protein FW) in intermediate SO_2_-tolerant* C. dactylon* accessions, and 3.4-fold (approximate 17 U/mg protein FW) in high SO_2_-tolerant* C. dactylon* accessions, respectively. More importantly, SiR activities showed significantly higher levels in leaves from high and intermediate SO_2_-tolerant* C. dactylon* accessions than those from SO_2_-sensitive* C. dactylon* accessions, displaying an apparent increasing trend along with SO_2_ tolerability of the* C. dactylon* accessions (Figure 7(b)). SO activities in leaves from any of* C. dactylon* accessions after 7-day SO_2_ fumigation treatment showed nearly the same level with those from 0-day time-point without SO_2_ treatment (Figure 7(c)). However, SO activity levels were significantly higher in leaves from high and intermediate SO_2_-tolerant* C. dactylon* accessions than those from SO_2_-sensitive* C. dactylon* accessions, displaying an apparent increasing trend along with SO_2_ tolerability of the* C. dactylon* accessions (Figure 7(c)).

## 4. Discussion

SO_2_, a major air pollutant in developing countries, is highly toxic to plants once they are exposed to high doses of SO_2_ above the threshold.* C. dactylon* is a widely used warm season turfgrass on sports fields, golf courses, roadsides, and lawns in city or industry districts. Our previous study indicated that growth rate of* C. dactylon* was affected and visible symptoms appeared on leaves under SO_2_ stress condition; however this species has much better SO_2_-tolerant ability among warm season turfgrasses [13].* C. dactylon* is wildly distributed in South America, Africa, Europe, and South Asia and displays abundant genetic diversities worldwide [30–33]. To achieve better air quality and landscape effect in SO_2_ polluted areas, selection or development of high SO_2_-tolerant* C. dactylon* variations for planting in such regions is desired. In this study, we selected 9 out of 38 wild* C. dactylon* accessions from Southwest China as representatives of high, intermediate SO_2_-tolerant, and SO_2_-sensitive accessions based on the injury degree of SO_2_ to leaves and the geographic distribution and then comparatively analyzed their physiological differences under SO_2_ untreated and treated conditions. Our results indicated that SO_2_ tolerance of* C. dactylon* might be largely related to soluble sugar, proline and chlorophyll a contents, and SO enzyme activities. To the best of our knowledge, this is the first comprehensive study of physiological differences in* C. dactylon* accessions of warm season turfgrasses. This study gained some insights into understanding the genetic and molecular SO_2_-tolerant mechanisms of* C. dactylon* and provided guideline for selection and development of* C. dactylon* variations for planting in SO_2_ polluted urban or industrial areas.

Soluble sugars and proline, as two major compatible solutes in the cytoplasm and organelle, play important roles under multiple stress conditions, such as drought and salinity [34, 35]. In this study, we observed that SO_2_-tolerant* C. dactylon* accessions showed significantly higher soluble sugar and proline contents under both SO_2_ treated and untreated conditions (Figure 3), suggesting that both of them might be related to SO_2_ tolerance.* C. dactylon* accessions originated from habitats of hillside and city park have much higher SO_2_ tolerance than those from other habitats (Tables 1 and 2), suggesting that the increased soluble sugar and proline contents most probably evolved from drought and SO_2_ stress adaptation. However, increased soluble sugar and proline contents in SO_2_-tolerant* C. dactylon* accessions are not likely involved in osmotic pressure but more likely involved in maintaining cell membrane stability, synthesis of other compounds, supply of energy, action as regulators of gene expression, and signal molecules based on their multiple functions [36]. Thereafter, soluble sugar and proline contents can be considered as marker for selection of* C. dactylon* variations with high SO_2_ tolerability.

Chlorophyll (including chlorophylls a and b) and carotenoid are known as the two important pigments in chloroplast of tree and grass plant leaves. The important role of pigments is to absorb certain wavelengths from sunlight and then convert the unusable sunlight energy into usable chemical energy during photosynthesis. Chlorophyll a is the primary pigment for photosynthesis in plants [37]. In this study, leaf injury of* C. dactylon* was observed under SO_2_ stress condition (Table 2). As a consequence, chlorophyll a, chlorophyll b, and carotenoid contents decreased in* C. dactylon* under SO_2_ stress condition, consistent with previous reports on grass and tree plants [13, 38, 39]. However, SO_2_-tolerant* C. dactylon* accessions showed significantly higher contents of chlorophyll a, chlorophyll b, and carotenoid under SO_2_ treated condition, consistent with their less leaf injury SO_2_-tolerant* C. dactylon* accessions observed in this study. Moreover, SO_2_-tolerant* C. dactylon* accessions had significantly higher content of chlorophyll a under SO_2_ untreated condition. Now that chlorophyll a is the primary pigment for photosynthesis in plants, significantly higher contents of chlorophyll a in* C. dactylon* accessions under both SO_2_ treated and untreated conditions indicate that SO_2_ tolerance of* C. dactylon* might be largely related to content of chlorophyll a.

Early study showed that SO_2_ gas after entering leaves of plant is converted into sulfite (SO_3_^2−^) and bisulfite (HSO_3_^2−^) once it is dissolved in cellular cytoplasm [40]. Furthermore, detoxification reaction of HSO_3_^2−^ and SO_3_^2−^ to sulfate (SO_4_^2−^) in plants leads to production of many kinds of ROS, such as superoxide radical (O2^−*∙*^), hydrogen peroxide (H_2_O_2_), and hydroxyl radical (OH^*∙*^) [41]. Excessive ROS are highly reactive and toxic to plants, which could cause oxidative damage to membranes, DNA, proteins, photosynthetic pigments, and lipids [42]. To protect plant cells from ROS damage, plant developed antioxidant enzymes to deal with the excessive ROS in plant cells. SOD, POD, and CAT are considered as three major antioxidant enzymes. To analyze the oxidative effect of SO_2_ on* C. dactylon*, we measured the ROS level and antioxidant enzyme activities in* C. dactylon* accessions. Although both ROS levels (reflected by H_2_O_2_ and MDA contents) and antioxidant enzyme activities (reflected by SOD, POD, and CAT) increased in all of the nine* C. dactylon* accessions under SO_2_ stress condition, the SO_2_-tolerant* C. dactylon* accessions showed significantly lower ROS levels and SOD activities, indicating that the SO_2_-tolerant* C. dactylon* accessions have much stronger antioxidant ability and less damage occurs to them by SO_2_. Moreover, lower SOD activity was theoretically consistent with lower ROS level in the SO_2_-tolerant* C. dactylon* accessions under SO_2_ stress condition, which is in agreement with previous report [43]. Although POD activities were nearly at the same level in leaves from all of the nine* C. dactylon* accessions after 7-day SO_2_ stress treatment, activities of this antioxidant enzyme from SO_2_-tolerant* C. dactylon* accessions were significantly higher than those from SO_2_-sensitive* C. dactylon* accessions. Taken together, we suggest that significantly higher activity of POD prior to SO_2_ treatment might be devoted to the increased antioxidant ability in SO_2_-tolerant* C. dactylon* accessions.

SO_2_ gas after entering leaves of plant can be converted into either sulfate by SO to enter into oxidative pathway or sulfide by SiR to enter into reductive pathway [44]. Overexpression of both SO and SiR showed more tolerance to sulfur dioxide toxicity in* Arabidopsis thaliana* and/or tomato plants [44–47]. Transcriptional analyses indicate that SiR is induced by SO_2_ but SO is constitutively expressed in natural plant [45, 46]. In this study, we found that SiR activity level was significantly increased under SO_2_ stress condition but SO activity level had almost no change under SO_2_ treated and untreated conditions in leaves from all of the nine* C. dactylon* accessions, consistent with previous reports on other plant species [45, 46]. Under SO_2_ stress condition, the SO_2_-tolerant* C. dactylon* accessions showed higher levels of both SiR and SO activities and contained higher sulfur content in leaves as corresponding consequence. More importantly, we found that the SO_2_-tolerant* C. dactylon* accessions showed significantly higher SO activities prior to SO_2_ treatment, but no significant differences were observed among the nine* C. dactylon* accessions. Increased SO activity in SO_2_-tolerant* C. dactylon* accession could convert sulfite to nontoxic sulfate more efficiently than SO_2_-sensitive* C. dactylon* accession for storage, once highly toxic SO_2_ gas enters into the* C. dactylon* cells, which indicates that SO antioxidant enzyme plays an important role in SO_2_ tolerance in* C. dactylon*.

## 5. Conclusion

*C. dactylon*, a warm season perennial grass species, is widely used as turfgrass on sports fields, golf courses, roadsides, and lawns in city or industry districts in warm season. Although this species has much better SO_2_-tolerant ability among warm season turfgrasses, its growth rate will be affected and visible symptoms like yellowing, chlorosis, bleaching, and even killing foliage will appear on leaves of* C. dactylon* in SO_2_ polluted areas. To achieve better air quality and landscape effect in SO_2_ polluted areas, selection or development of high SO_2_-tolerant* C. dactylon* variations is desired. In this study, we selected 9 out of 38* C. dactylon* accessions from Southwest China as representatives of high, intermediate SO_2_-tolerant, and SO_2_-sensitive accessions and then comparatively analyzed their physiological differences under SO_2_ untreated and treated conditions. Our results indicated that SO_2_ tolerance of* C. dactylon* might be largely related to soluble sugar, proline and chlorophyll a contents, and SO enzyme activities. This study gained some insights into understanding the genetic and molecular SO_2_-tolerant mechanisms of* C. dactylon* and provided guideline for selection or development of* C. dactylon* variations for planting in SO_2_ polluted urban or industrial areas.

## Figures and Tables

**Table 1:** Geographical origin of 38 wild *C. dactylon* accessions used in this study.

Order	Accession number	Origin	Habitat	Altitude (m)	Mean annual temperature (°C)
1	SC1102	Pixian, Sichuan	Roadside	560	18.5
2	SC1105	Longquan, Sichuan	Hillside	750	16.5
3	SC1106	Shuangliu, Sichuan	Riverside	510	16.3
4	SC1109	Guangyuan, Sichuan	Roadside	490	16.1
5	SC1115	Zitong, Sichuan	Roadside	610	16.5
6	SC1119	Guanghan, Sichuan	Riverside	420	16.3
7	SC1201	Leshan, Sichuan	Floodland	420	17.4
8	SC1203	Wuhou, Sichuan	City park	540	16.7
9	SC1208	Luxian, Sichuan	Riverside	380	17.8
10	SC1209	Nanchong, Sichuan	Hillside	520	17.4
11	SC1211	Guang'an, Sichuan	Roadside	450	17.1
12	SC1213	Panzhihua, Sichuan	Roadside	1150	20.3
13	SC1217	Meishan, Sichuan	Fieldridge	460	17.1
14	CQ1101	Jiangjin, Chongqing	Roadside	590	18.4
15	CQ1102	Yongchuan, Chongqing	Floodland	340	18.2
16	CQ1107	Bishan, Chongqing	Roadside	530	18.3
17	CQ1108	Jiangbei, Chongqing	Roadside	440	17.5
18	CQ1109	Dianjiang, Chongqing	Fieldridge	420	17.0
19	CQ1112	Liangping, Chongqing	Roadside	520	16.6
20	CQ1116	Wanzhou, Chongqing	Riverside	180	17.7
21	YN1102	Kunming, Yunnan	City park	1910	15.0
22	YN1105	Chuxiong, Yunnan	Roadside	1790	15.8
23	YN1106	Dali, Yunnan	Roadside	1980	15.1
24	YN1107	Lijiang, Yunnan	Riverside	2360	15.8
25	YN1110	Baoshan, Yunnan	Wasteland	2410	16.0
26	YN1201	Yuxi, Yunnan	Roadside	1890	18.2
27	YN1205	Pu'er, Yunnan	Roadside	1750	17.7
28	YN1208	Xishuangbanna, Yunnan	Roadside	890	21.0
29	GZ1103	Guiyang, Guizhou	Riverside	970	15.3
30	GZ1104	Anshun, Guizhou	Roadside	860	14.0
31	GZ1106	Liupanshui, Guizhou	Fieldridge	1810	13.5
32	GZ1109	Zunyi, Guizhou	Roadside	960	15.1
33	GZ1110	Qianlan, Guizhou	Hillside	1050	16.1
34	XZ1205	Lhasa, Tibet	Roadside	3120	7.5
35	XZ1206	Nyingchi, Tibet	Roadside	3430	8.7
36	XZ1208	Bomi, Tibet	Riverside	2680	8.7
37	XZ1209	Baxoi, Tibet	Roadside	3250	10.4
38	XZ1213	Chamdo, Tibet	Floodland	3170	7.6

**Table 2:** Leaf injury rate of 38 wild *C. dactylon* accessions under SO_2_ stress condition.

Order	Accession number	Injury rate (%)
1	YN1205	38.3 ± 0.6
2	CQ1116	37.7 ± 1.2
3	SC1208	36.7 ± 0.6
4	SC1106	35.3 ± 1.5
5	GZ1109	34.3 ± 1.5
6	YN1107	32.0 ± 1.7
7	YN1208	31.7 ± 0.6
8	SC1213	30.3 ± 1.5
9	CQ1112	30.3 ± 1.2
10	XZ1209	28.7 ± 1.2
11	XZ1213	28.3 ± 0.6
12	GZ1104	27.3 ± 1.5
13	SC1119	26.3 ± 1.5
14	SC1201	26.3 ± 2.1
15	YN1106	25.7 ± 1.5
16	XZ1205	25.7 ± 2.5
17	CQ1102	25.3 ± 0.6
18	SC1217	25.3 ± 1.5
19	YN1110	25.0 ± 1.0
20	XZ1206	24.7 ± 0.6
21	SC1211	24.7 ± 2.5
22	YN1105	23.7 ± 2.1
23	XZ1208	23.0 ± 1.7
24	GZ1106	22.0 ± 1.0
25	SC1102	21.7 ± 2.1
26	SC1115	20.0 ± 2.6
27	CQ1108	20.0 ± 2.6
28	CQ1109	19.3 ± 1.5
29	YN1201	19.3 ± 0.6
30	SC1109	19.3 ± 1.5
31	GZ1103	19.0 ± 2.0
32	CQ1107	18.7 ± 1.5
33	CQ1101	17.7 ± 1.5
34	YN1102	17.3 ± 1.5
35	SC1105	16.3 ± 1.5
36	GZ1110	15.0 ± 2.0
37	SC1209	13.7 ± 0.6
38	SC1203	13.3 ± 1.5

Note: data are presented by means ± SE (*n* = 3).
